# Levels of substitution of inorganic mineral to amino acids complexed minerals on old laying hens

**DOI:** 10.1038/s41598-024-75897-x

**Published:** 2024-10-22

**Authors:** Marcos J B Santos, Carlos B V Rabello, Jamille S S Wanderley, Maria C M M Ludke, Mércia R Barros, Fabiano S Costa, Clariana S Santos, Alba K Fireman

**Affiliations:** 1 Department of Animal Science, Rural Federal University of Pernambuco, Recife, PE Brazil; 2 Department of Veterinary Science, Rural Federal University of Pernambuco, Recife, PE Brazil; 3 Zinpro corporation, Eden Prairie, MN 55344 USA

**Keywords:** Metal-amino acids, Radiodensity, Trace minerals, Metals, Animal physiology

## Abstract

This study was conducted with the objective of evaluating the impact of replacing inorganic mineral sources (IM) with amino acid complexed minerals (AACM) in laying hens’ diets on performance, egg quality, bone, and intestinal health. The effects of 4 different diets with varying levels of AACM substitution were evaluated on 400 Lohmann White hens aged 78–98 weeks. The control diet contained only IM sources at levels of 60, 60, 7, 40, 0.2, and 2 mg/kg of Zn, Mn, Cu, Fe, Se, and I, respectively. The other treatments were made by a total substitution of IM with AACM, as follows: AACM70–70% of IM levels; AACM50–50% of IM levels; and AACM40–40% of IM levels. Orthogonal polynomial contrasts and Dunnett’s test were used to determine their impact (*P <* 0.05). The treatment AACM40 improved egg production, egg weight, egg mass, and feed conversion ratio (*P* < 0.05). Hens that received AACM40 also produced the thickest eggshells and better tibial bone density (*P <* 0.01). Histomorphometry analyses demonstrated significant effects of AACM treatments. The optimal supplementation levels of 24, 24, 2.8, 16, 0.08, and 0.8 mg/kg of Zn, Mn, Cu, Fe, Se, and I, respectively.

## Introduction

The body mineral composition of poultry is comprised of trace minerals including zinc (Zn), manganese (Mn), copper (Cu), iron (Fe), and selenium (Se), as well as macro minerals such as calcium (Ca) and phosphorus (P). These minerals are distributed throughout various anatomical structures within the poultry organism, including the liver, pancreas, and spleen, with a predominant accumulation observed in the tibias^1,2^.

Trace minerals are essential in poultry diets as they participate in bone metabolism, enzymatic reactions, hormones, immune system function, tissue synthesis, and other physiological functions^1,3,4^. To prevent deficiencies, these nutrients should be supplemented in diets at optimal levels that meet the animal’s mineral requirements. However, factors such as bioavailability, mineral interactions, and the presence of dietary antagonists should be considered when determining inclusion rates. In addition, excessive mineral supplementation can also be detrimental, leading to decreases in zootechnical performance and environmental pollution^1^.

While trace minerals are present in basal diets, current production standards often require additional supplementation of trace minerals to support optimal animal performance. Most commonly, inorganic minerals (IM), like oxides and sulfates, are added to the diet to meet the animals’ requirements. Different IM sources can have different bioavailability^4^ and all IM sources face certain obstacles to absorption within the digestive tract. Dietary chelators like phytate, folic acid, and tannins are known to bind to free mineral ions, creating insoluble complexes that make the mineral unavailable to the animal^5,6^. Additionally, minerals can have antagonistic effects towards one another and compete for the same absorption pathways; for example, both Cu and Fe can use the divalent metal transporter 1 (DMT1) for absorption and so compete for access to these transporters^5^. With such challenges to absorption, IM supplementation is not always the most efficient choice.

An alternative to IM sources are organic trace minerals, mineral sources where the active metal ion is complexed or chelated to an organic ligand molecule. The bioavailability and stability of organic trace minerals depend on the chelating agent used. Thus, the chelating agent must be carefully selected to achieve an optimal balance. Some organic trace minerals show no benefits over supplementation with IM sources, Muhammad et al.^7^ found no differences in egg production between inorganic Na_2_SeO_3_ and Se-yeast in laying hens. Some studies have found inferior results with chelated minerals compared to IM. Rostagno et al.^8^ showed that yeast-bound trace minerals had 56% lower bioavailability in birds compared to IM sources. Kim et al.^9^ also reported improved results with IM in comparison with soy-protein-bound minerals for Cu, Mn, Zn, and Fe supplementation. Conflicting evidence indicates that the efficacy of minerals bound to an organic molecule depends on the specific mineral, ligand used, and physiological state.

Trace minerals complexed to amino acids (AACM) at a 1:1 ratio have been investigated in poultry nutrition to address bioavailability concerns^1,10,11^. Unlike IM, AACM does not interact with other compounds due to the stability of the mineral amino acid complex, which protects them from dietary chelators. Medeiros-Ventura et al.^12^ indicated that compared to IM, AACM can enhance mineral bioavailability and absorption, thereby improving the trace mineral status and performance of broilers and layers. The AACM uses active transport mechanisms facilitated by their amino acid ligands for absorption^6^. Furthermore, these characteristics indicate that AACM supplementation levels can be lower than IM sources, and the inclusion rates required for AACM may help mitigate the toxicity risks associated with high IM levels.

Due to higher production in modern laying hens as they age, several challenges can compromise their performance. These include lower intestinal absorption, elevated stress levels from long-term confinement, increased mineral requirements to maintain egg size, and osteoporosis caused for higher demand for minerals to eggshell formation^13^. Sun et al.^14^ found a direct correlation between eggshell thickness and egg breakage resistance. Also, Baur et al.^15^ evaluated keel bone injuries in laying hens. They discovered that 99% of the chickens had at least one injury, with 97% suffering fractures, with an average of 3.09 fractures per keel bone, revealing a significant welfare problem in commercial farms. The depletion of essential trace minerals such as Zn, Cu, Mn, and Se is particularly concerning. These minerals play crucial roles in various physiological processes, including enzyme function, antioxidant defense, and bone health^5^. In this way, a correct supplementation of trace minerals with a more bioavailable source can be an effective approach to mitigate the challenges associated with aging laying hens in intensive production systems. Continued research is important to refine our understanding of trace mineral nutrition and establish updated recommendations for laying hens. With proper supplementation, trace minerals support health, growth, and productivity in commercial poultry production.

Therefore, we aimed to evaluate the effect of the total replacement of Zn, Mn, Cu, Fe, and Se AACM in substitution of IM sources in laying hen diets on performance, egg quality, bone, and intestinal variables. We hypothesize that the complete replacement of IM with AACM at reduced inclusion levels will improve laying hen performance, egg quality, bone health, and intestinal morphology.

## Materials and methods

### Animal ethics statement

This study was conducted in accordance with the guidelines of the Ethics Committee on Animal Use at the Federal Rural University of Pernambuco, approved under No. 041/2016 and all animal experiments complied with the ARRIVE guidelines.

### Animals and husbandry

A total of 400 laying hens acquired from a commercial farm, Lohmann White strain, aged 78 to 98 weeks, were housed in cages measuring 100 × 40 × 45 cm, with 10 replicates and 10 hens per cage, providing 450 cm² per hen. The cages were equipped with trough-type feeders and nipple drinkers. The experimental period lasted 140 days, feed and water were available ad libitum throughout the experimental period.

The environmental conditions inside the facility were monitored daily, with an average temperature of 27ºC and relative humidity of 81%, using a digital thermo-hygrometer and datalogger (HOBOware; U12-012, Onset Computer Corporation, Bourne, MA, USA). The lighting program adopted consisted of 16 h a day (natural light + artificial light).

### Experimental design and dietary treatments

The 4 treatments consisted of an IM source, and a complete replacement of IM in with AACM at 70%, 50%, or 40% of the trace mineral requirement based on the Lohmann White strain guide^16^. Diets were categorized as follows:

- IM - diet containing 60, 60, 7, 40, 0.2, and 2 mg/kg of Zn, Mn, Cu, Fe, Se, and I, respectively;
- AACM70 - 42, 42, 4.9, 28, 0.14, and 1.4 mg/kgof Zn, Mn, Cu, Fe, Se, and I, respectively;
- AACM50 - 30, 30, 3.5, 20, 0.10, and 1 mg/kg of Zn, Mn, Cu, Fe, Se, and I, respectively;
- AACM40 - 24, 24, 2.8, 16, 0.08, and 0.8 mg/kgof Zn, Mn, Cu, Fe, Se, and I, respectively. Diet composition is presented in Table 1, and Table 2 describes the chemical composition of the diets. Zinc oxide, Mn oxide, Cu sulfate, Fe sulfate, sodium selenite, and calcium iodate were used as IM. The AACM minerals were sourced from Zinpro^®^ Performance Minerals^®^. The trace microminerals Zn, Mn, Cu, and Fe were complexed with a non-specific essential amino acid ligand in 1:1 ratio. Additionally, iodine was combined with the Zn molecule, and Se was provided in the form of Zn-L-selenomethionine.

**Table 1:** Ingredients and calculated nutrient content of the basal diet.

Ingredients^1^	content
Ingredients, g/kg as-fed basis	
Corn	597
Soybean meal	250
Soybean oil	17.0
Limestone	109
Dicalcium phosphate	9.20
Sodium bicarbonate	1.50
Salt	2.90
DL-Methionine, 99%	2.90
L-Threonine, 98.5%	0.50
Phytase ^2^	0.06
Premix Vitamin ^3^	1.00
Premix Mineral^4^	1.50
Inert (sand)	6.40
Absorbent^5^	1.00
Total	1000
Nutritional Composition, g/kg as-fed basis	
AME, MJ/kg	11.5
Crude protein	161
Calcium	45.0
Phosphorus	6.40
Available phosphorus	4.10
Digestible lysine	7.60
Digestible methionine	5.30
Digestible Met + Cys	7.40
Digestible threonine	5.90

^1^Diets were formulated based on the recommendation of Lohmann White strain.

^2^Supplementation per kilogram of the product: Quantum Blue AB Vista phytase, ≥ 10,000 FTU/kg.

^3^Supplementation per kilogram of the product: Vitamin A, 8000 IU; Vitamin D3, 2000 IU; Vitamin E, 10,000 IU; Vitamin K3, 2000 mg; Vitamin B1, 1000 mg; Vitamin B2, 4000 mg; Vitamin B6, 2500 mg; Vitamin B12, 11,000 mg; Niacin, 25 g; Calcium pantothenate, 10 g; Folic acid, 550 mg; Biotin, 50 mg.

^4^ The premix minerals composition is described in Table 2.

^5^ Supplementation per kilogram of the product: Hydrated sodium and calcium aluminosilicates: 0.10 mg/g.

**Table 2:** Mineral compositions of experimental diets and water.

Treatments	Zn	Mn	Cu	Fe	Se	I^1^	Ca	P
	mg/kg						g/kg	
	Calculated							
IM	60	60	7.0	40	0.20	2.0	45	4.1
AACM70	42	42	4.9	28	0.14	1.4	45	4.1
AACM50	30	30	3.5	20	0.10	1	45	4.1
AACM40	24	24	2.8	16	0.08	0.80	45	4.1
		Analyzed^2^						
IM	62.3	67.3	9.30	462	0.31	-	45.0	6.01
AACM70	44.5	48.8	6.31	357	0.31	-	46.0	5.95
AACM50	33.9	34.1	4.25	266	0.29	-	47.0	5.95
AACM40	21.1	27.9	3.94	222	0.29	-	43.7	5.29
Watter	< 0.01	0.005	< 0.01	< 0.005	< 0.01	-	9.99	< 0.01

^1^Ca(IO_3_)_2_; mg/kg.

IM = inorganic minerals; AACM = amino acid complexed minerals^2^. Obtained by inductively coupled plasma source, dry matter basis. The amino acid complex sources were Zinpro Availa Zn, Mn, Cu, Fe, and Se (Zinpro Corporation., Eden Prairie, MN, USA);

### Mineral concentrations in the diets

During the experiment, feed samples were collected and analyzed for mineral composition. For this purpose, 0.5 g from each sample were weighed and digested in 6 mL of 65% HNO_3_ in a microwave oven (Mars Xpress: Technology Inside, CEM Corporation) for 30 min at 160 ºC. The resulting solution was filtered through quantitative blue stripe filter paper and diluted with deionized water to reach a volume of 25 ml. The quantification of minerals (Zn, Mn, Cu, Fe, Se, Ca, and P) in the samples was performed by inductively coupled plasma optical emission spectrometry (Optima 7000 DV ICP-OES, PerkinElmer). Water samples were collected in plastic containers and frozen. Quantification of minerals in the water was also performed by ICP-OES.

### Performance and measurements

During the experimental period, the performance and quality of the egg variables were analyzed. At the end of the experiment, one bird was selected from each experimental unit. Subsequently, the laying hens were humanely euthanized via cervical dislocation, and their intestines and tibias were collected for analysis.

Eggs were collected and weight daily, the following variables were evaluated: egg production (%), average daily feed intake (g/hen/ day), egg weight (g), egg mass (g/hen/day), and feed conversion ratio (g/hen/day and g/dozen eggs). Eggs were collected once daily. All produced eggs were counted and weighed, and feed leftovers were also weighed.

### Egg Quality

Each 28-day period, 30 eggs were collected over 3 days (3 eggs per experimental unit), to evaluate egg quality variables, including egg weight, yolk color, albumen height (mm), albumen weight (g), yolk weight (g), eggshell weight (g), eggshell thickness (mm), percentages of yolk, albumen, and eggshell, and the Haugh unit (HU).

To determine albumen height, eggs were cracked and their contents placed on a flat, level surface. Albumen heights were measured using a digital caliper (iGaging, San Clemente, CA, USA). The HU was calculated using the formula described by Card and Nesheim^17^, :

HU = 100 x log(AH + 7.57–1.7 x EW^0.37^).

where AH = albumen height (mm), and EW = egg weight (g).

Subsequently, the yolks were separated from the albumen and weighed on a precision balance. The eggshells were washed, dried at room temperature (27 ºC) for 48 h, and weighed. Using a digital caliper (iGaging, San Clemente, CA, USA), eggshell thickness was measured by selecting 2 distinct points in the center-transverse area. Yolk, albumen, and eggshell percentages were calculated using their respective weights in relation to egg weight. Colorimetric analysis of the yolk was performed using a color fan, on a scale from 1 to 15 (DSM).

### Egg yolk collection and evaluation

Egg yolks were collected and placed in plastic bags. At the end of each period, a pool was created, combining 2 yolks per replication. The material was then dried in an oven at 105ºC for 12 h. After drying, the material was crushed and 0.5 g of it was weighed. The digestion process was performed using 6 ml of concentrated HNO_3_ in a microwave (MarsXpress - CEM Technology) at 160 ºC for 35 min. Upon completion of the digestion process, deionized water was added to the samples to reach a total volume of 25 ml. Following the protocol described by Ramos et al.^18^, the samples were then filtered using quantitative filter paper and further diluted to a total volume of 25 ml. The quantification of minerals in the water was performed by ICP-OES.

### Bone variables

After euthanasia, the right and left tibias were collected. The left tibias were weighed and dried in an oven at 105°C for 24 h, followed by calcination in a muffle furnace (model 2000 F; Zezimaq, Minas Gerais, Brazil) for 4 h at 600°C. Subsequently, a 0.5 g sample was weighed (± 0.0001 g) and digested in 6 mL of HNO_3_ (65% P.A.) for 10 min in an open system. In addition, it was diluted in 25 mL of deionized water and the mineral composition was quantified by ICP-OES. The right tibias were weighed and measured using a digital caliper to determine the Seedor index, calculated by dividing the ash weight (mg) by the bone length (mm)^19^.

Bone densitometry was performed on 5 right tibias per treatment using Hi Speed FXI CT scanner equipment (General Electric, Fairfield, CT 06824, USA). Cross-sectional images were acquired from 2-mm-thick sections with a reconstruction interval of 1 mm. These images were analyzed using Dicom software (version 1.1.7. Horos, Purview, Annapolis, MD 21401, USA) to estimate individual bone radiodensity values at the 3 diaphysis cut levels (proximal, medial, and distal). A circular region of interest was selected for assessing cortical bone densitometry^20^.

### Intestine histology

After euthanasia, the small intestine was collected and weighed, and its segments (duodenum, jejunum, and ileum) were preserved in airtight containers with 10% formaldehyde solution. Subsequently, they were immersed in xylol before being embedded in paraffin^21^.

One slide was produced per animal per intestinal section, with 3 cuts for each intestinal portion, totaling 120 slides (3 slides x 4 treatments x 10 replicates), all of which were stained with hematoxylin-eosin. Photomicrographs were captured, and the images were subsequently analyzed using ImageJ software. The variables under analysis included villus height and width, crypt depth and width, villus: crypt ratio, and villus surface area. For each segment and variable, 12 measurements were recorded, resulting in a total of 72 measurements per treatment. To assess the behavior of the absorptive surface of the segments, the formula proposed by Kisielinski et al.^22^ was applied.

$$M=\frac{\left(villus\,width\,\times\,villus\,height\right)+\left(\frac{villus\,width}2+\,\frac{crypt\,width}2\,\right)^2\times\,\left(\frac{villus\,width}2\,\right)^2}{\left(\frac{villus\,width}2+\frac{crypt\,width}2\,\right)^2}$$

Where M is the surface area of the villus.

### 10 statistical analysis

The normality and homoscedasticity assumptions were tested for analysis of variance. Orthogonal polynomial contrasts were used to assess linear and quadratic trends across AACM treatment levels (AACM40, AACM50, and AACM70). Dunnett’s test was used to compare each AACM treatment group (AACM40, AACM50, and AACM70) to the IM control group. Differences were considered significant at *P* < 0.05 for both orthogonal polynomial contrasts and Dunnett’s test. Version 9.4 of SAS software was utilized^23^. The following statistical model was used:

Y_ij_ = µ + τ_i_ + ϵ_ij_.

Where:

- Y_ij_ represents the dependent variable.
- µ is the overall mean.
- τ_i_ is the effect of the i^th^ treatment which corresponds to one of the specific dietary treatments (IM, AACM70, AACM50, or AACM40) given to the hens.
- ϵ_ij_ is the random error associated with each observation.

## Results

### Performance

Dietary treatments influenced the performance variables of laying hens (Table 3). No differences in average daily feed intake were observed between treatments (*P =* 0.15). However, there was a linear decrease in feed intake (*P =* 0.05) as the AACM inclusion rate reduced. Egg production was 3%, 5%, and 7% higher for hens receiving AACM70, AACM50, and AACM40, respectively, compared to the IM control (*P =* 0.02). Additionally, a linear trend was noted in egg production across AACM treatments (*P =* 0.04). A similar pattern was observed for egg weight and egg mass. The AACM40 treatment resulted in heavier eggs compared to IM-supplemented hens. On average, eggs from AACM40 hens were 1.7% heavier than IM eggs (*P =* 0.03). This resulted in superior egg masses of 5.9% and 9.7% for AACM50 and AACM40, respectively, relative to IM (*P =* 0.04). However, AACM70 did not differ from the IM group. A linear improvement in egg weight and egg mass was observed as AACM content decreased in the treatment groups (*P =* 0.01). In terms of FCR, the AACM inclusion resulted in significant improvements. Compared with AACM40 hens, the IM group required 207 g more feed to produce 1 kg of eggs (*P <* 0.01). Likewise, the gradual inclusion in AACM content showed a significant linear trend in feed conversion ration improvement (*P <* 0.01). To produce a dozen eggs, similar results were observed for hens supplemented with AACM40, which required 116 g less feed when compared to those fed IM, although this was not different from other treatments. Nonetheless, a linear trend was observed with a decrease in AACM content in the diet on feed conversion ratio (*P <* 0.01).

**Table 3:** Performance of white laying hens fed different sources and levels of Zn, Mn, Cu, Fe, Se, and I from 78 to 98 weeks of age.

Treatments	ADFI, g	EP, %	EW, g	EM, g	FCR, g/g	FCR, g/dozen eggs
IM	105	73.6*	67.9*	50.0*	2.075*	1.671*
AACM70	105	76.7*	67.9	52.0	2.012	1.645
AACM50	104	78.6*	68.1	53.1*	1.967	1.607
AACM40	103	80.8*	69.1*	55.3*	1.868*	1.555*
MEAN	104	77.1	68.3	52.6	1.960	1.610
SEM	0.37	0.78	0.17	0.57	0.0161	0.0122
		*P*-values				
Dunnett	0.154	0.022	0.026	0.004	0.001	0.023
Linear^1^	0.054	0.044	0.011	0.014	< 0.001	< 0.001
Quadratic^1^	0.674	0.801	0.275	0.605	0.403	0.722

ADFI = average daily feed intake; EP = egg production; FCR = feed conversion ratio for egg mass; FCRD = feed conversion for dozen eggs; SEM = standard error of the mean.

IM = 60, 60, 7, 40, 0.2, and 2 mg/kg of Zn, Mn, Cu, Fe, Se, and I, respectively;

AACM70 = 42.0, 42.0, 4.9, 28, 0.14, and 1.4 mg/kg of Zn, Mn, Cu, Fe, Se, and I, respectively;

AACM50 = 30, 30, 3.5, 20, 0.10, and 1 mg/kg of Zn, Mn, Cu, Fe, Se, and I, respectively;

AACM40 = 24, 24, 2.8, 16, 0.08, and 0.80 of Zn, Mn, Cu, Fe, Se, and I, respectively^1^. Orthogonal polynomial contrast; *Within a column differ significantly (*P* < 0.05) according to Dunnett’s test, which compares AACM levels with the IM control group.

### Egg quality

Table 4 presents the egg quality variables. No differences were observed in albumen height (*P =* 0.08), % albumen (*P =* 0.07), % yolk (*P =* 0.06), Haugh unit (*P =* 0.09), and yolk color (*P =* 0.87) between the IM and AACM groups. Additionally, the orthogonal contrast test did not reveal any linear or quadratic trends for albumen height (*P =* 0.12), albumen (*P =* 0.96), Haugh unit (*P =* 0.07), or yolk color (*P >* 0.05). However, hens that received AACM40 produced the thickest eggshells (*P* < 0.01), with an average thickness 4.2% higher than IM shells. Furthermore, incremental improvements were also observed in eggshell thickness, demonstrating a linear response (*P <* 0.01) to reduced AACM supplementation. Both % yolk and % eggshell displayed a quadratic effect (*P =* 0.04 and *P =* 0.02, respectively), with AACM50 having the highest mean value.

**Table 4:** Egg quality from white laying hens fed different sources and levels of Zn, Mn, Cu, Fe, Se, and I from 78 to 98 weeks of age.

Treatments	AH, mm	ST, mm	A, %	Y, %	S, %	Haught unit	Color
IM	8.20	0.407*	60.8	27.4	9.31*	88.5	5.19
AACM70	8.42	0.410	61.4	26.7	9.14*	89.6	5.20
AACM50	8.56	0.419	61.5	27.4	9.31	90.4	5.15
AACM40	8.38	0.424*	61.5	26.8	9.13*	89.2	5.16
MEAN	8.39	0.415	61.3	27.1	9.31	89.4	5.18
SEM	0.05	0.0021	0.12	0.11	0.032	0.28	0.023
		*P-*values					
Dunnett	0.082	0.005	0.070	0.065	0.052	0.092	0.872
Linear^1^	0.716	0.003	0.919	0.642	0.863	0.504	0.520
Quadratic^1^	0.120	0.623	0.964	0.039	0.020	0.071	0.648

AH = albumen height; ST = eggshell thickness; A = albumen; Y = yolk; S = eggshell.

SEM = standard error of the mean.

IM = 60, 60, 7, 40, 0.2, and 2 mg/kg of Zn, Mn, Cu, Fe, Se, and I, respectively;

AACM70 = 42.0, 42.0, 4.9, 28, 0.14, and 1.4 mg/kg of Zn, Mn, Cu, Fe, Se, and I, respectively;

AACM50 = 30, 30, 3.5, 20, 0.10, and 1 mg/kg of Zn, Mn, Cu, Fe, Se, and I, respectively;

AACM40 = 24, 24, 2.8, 16, 0.08, and 0.80 of Zn, Mn, Cu, Fe, Se, and I, respectively^1^. Orthogonal polynomial contrast; *Within a column differ significantly (*P* < 0.05) according to Dunnett’s test, which compares AACM levels with the IM control group.

### Egg mineral deposition

**Figure 1:**
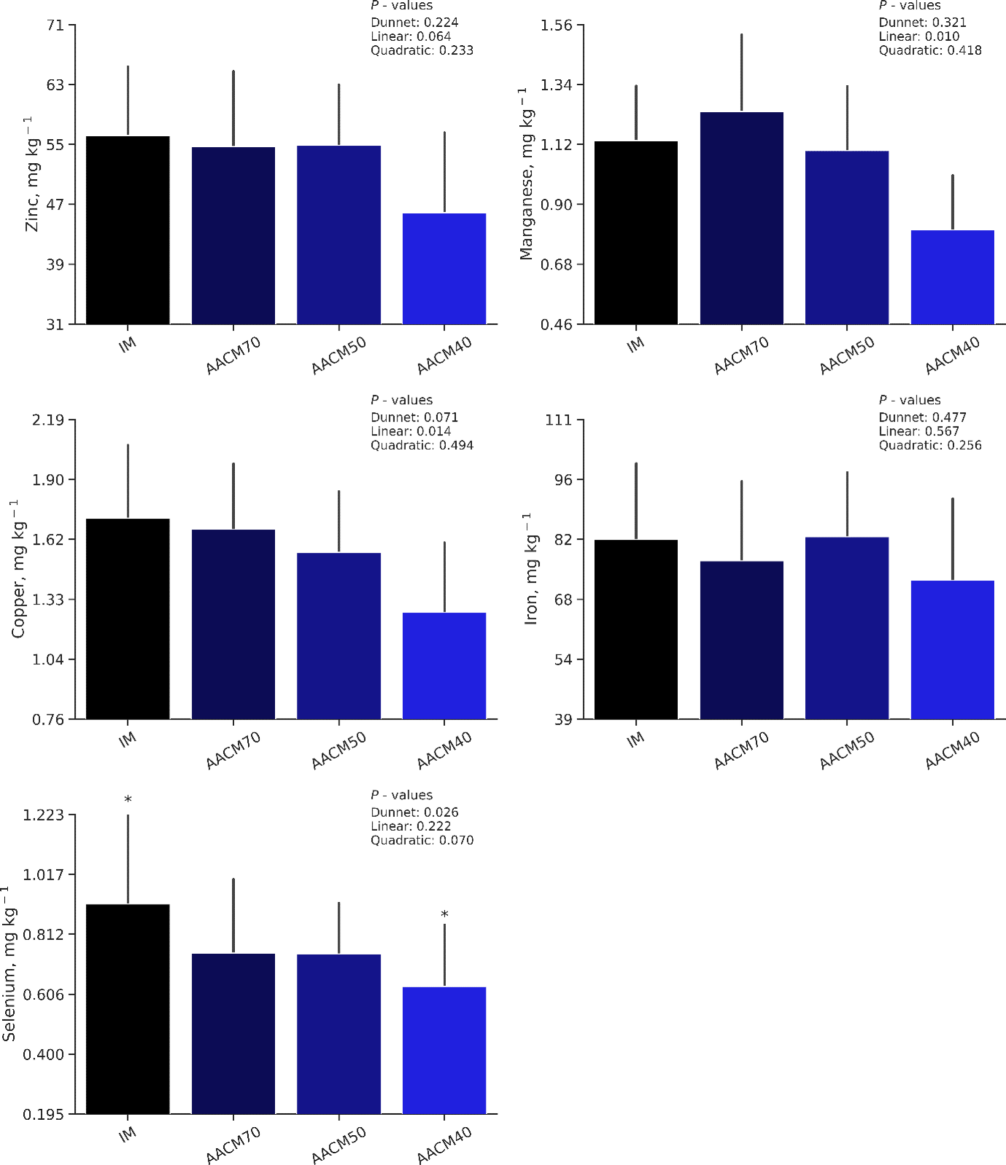
Egg yolk mineral deposition of trace minerals in eggs from laying hens fed different sources and levels of Zn, Mn, Cu, Fe, Se, and I from 78 to 98 weeks of age (mg/kg of dry yolk).  IM = 60, 60, 7, 40, 0.2, and 2 mg/kg of Zn, Mn, Cu, Fe, Se, and I, respectively;  AACM70 = 42.0, 42.0, 4.9, 28, 0.14, and 1.4 mg/kg of Zn, Mn, Cu, Fe, Se, and I, respectively;  AACM50 = 30, 30, 3.5, 20, 0.10, and 1 mg/kg of Zn, Mn, Cu, Fe, Se, and I, respectively; AACM40 = 24, 24, 2.8, 16, 0.08, and 0.80 of Zn, Mn, Cu, Fe, Se, and I, respectively. *Differ significantly by Dunnett’s test ( P <  0.05).

Egg mineral deposition is shown in Fig. 1. There were no differences between IM and AACM treatments for Zn (*P =* 0.22), Mn (*P =* 0.32), Cu (*P =* 0.07), and Fe (*P =* 0.47). However, AACM40 exhibited a lower Se content in egg yolk compared to IM treatment (*P =* 0.03). The orthogonal contrasts indicated no significant variations in egg Zn, Fe, or Se content between treatments (*P >* 0.05). However, there was a linear decrease in egg Mn content (*P <* 0.01) with decreasing AACM supplementation. The AACM40 treatment exhibited the lowest Mn content, which was 35% lower than that of the AACM70 treatment. Similarly, egg Cu deposition showed a linear decrease (*P =* 0.01) as dietary AACM supplementation reduced, with the lowest level recorded in the AACM40 compared to the AACM70 group.

### Tibia variables

Regarding tibia variables, tibia weight (*P =* 0.09), tibia length (*P =* 0.72), Seedor index (*P =* 0.20), and bone strength (*P =* 0.33) did not differ between the IM control and AACM supplementation groups at any inclusion level (Table 5). Furthermore, not significant effects on ash weight (*P =* 0.58), % ash (*P =* 0.82), Ca content (*P =* 0.19), phosphorus content (*P =* 0.18), or Ca/P ratio (*P =* 0.12) were observed. When examining the contrasts, both linear and quadratic analyses did not reveal differences among the treatments for tibia weight (*P >* 0.05), tibia length (*P >* 0.05), Seedor index (*P >* 0.05), or bone strength (*P >* 0.05). This indicates a lack of dose-dependent trends for these variables. There were no linear or quadratic trends on ash weight or percentage (*P >* 0.05). However, Ca content in the tibias exhibited a quadratic trend (*P =* 0.05), with the AACM50 treatment showing a 13% higher mean value than the AACM70 treatment and a 20% higher mean value compared to AACM40. Phosphorus content also displayed a quadratic trend (*P =* 0.05), with the AACM50 treatment being 13.2% higher than AACM70 and 21% higher than AACM40. The Ca/P was unaffected by the treatments (*P >* 0.05).

**Table 5:** Tibia weight, tibia length, Seedor index, tibia strength, ash, calcium (ca), phosphorus (P), and ca: P ratio of laying hens fed different sources and levels of Zn, Mn, Cu, Fe, Se, and I from 78 to 98 weeks of age.

Treatments	Tibia weight	Tibia length	Seedor Index	Tibia strength	Ashes (g)	Ashes (%)	Ca (mg/kg)	*P* (mg/kg)	Ca: *P* ratio
	(mg)	(mm)		(*N*)					
IM	7727	114	68.2	259	2.56	34.3	176	79.9	2.21
AACM70	7621	114	70.7	284	2.64	35.2	169	75.8	2.23
AACM50	8127	113	71.8	306	2.74	34.9	195	87.4	2.23
AACM40	8229	114	72.1	276	2.73	34.0	156	69.3	2.26
Average	7918	114	70.7	281	2.66	34.6	174	77.9	2.23
SEM	102.2	0.28	0.74	9.3	0.049	0.47	6.4	3.01	0.007
*P-*values									
Dunnett	0.092	0.718	0.201	0.331	0.580	0.822	0.192	0.177	0.124
Linear^1^	0.149	0.472	0.073	0.072	0.520	0.393	0.364	0.333	0.172
Quadratic^1^	0.201	0.710	0.691	0.919	0.683	0.818	0.052	0.053	0.435

SEM = standard error of the mean.

IM = 60, 60, 7, 40, 0.2, and 2 mg/kg of Zn, Mn, Cu, Fe, Se, and I, respectively;

AACM70 = 42.0, 42.0, 4.9, 28, 0.14, and 1.4 mg/kg of Zn, Mn, Cu, Fe, Se, and I, respectively;

AACM50 = 30, 30, 3.5, 20, 0.10, and 1 mg/kg of Zn, Mn, Cu, Fe, Se, and I, respectively;

AACM40 = 24, 24, 2.8, 16, 0.08, and 0.80 of Zn, Mn, Cu, Fe, Se, and I, respectively^1^. Orthogonal polynomial contrast.

### Tibia bone density

**Figure 2:**
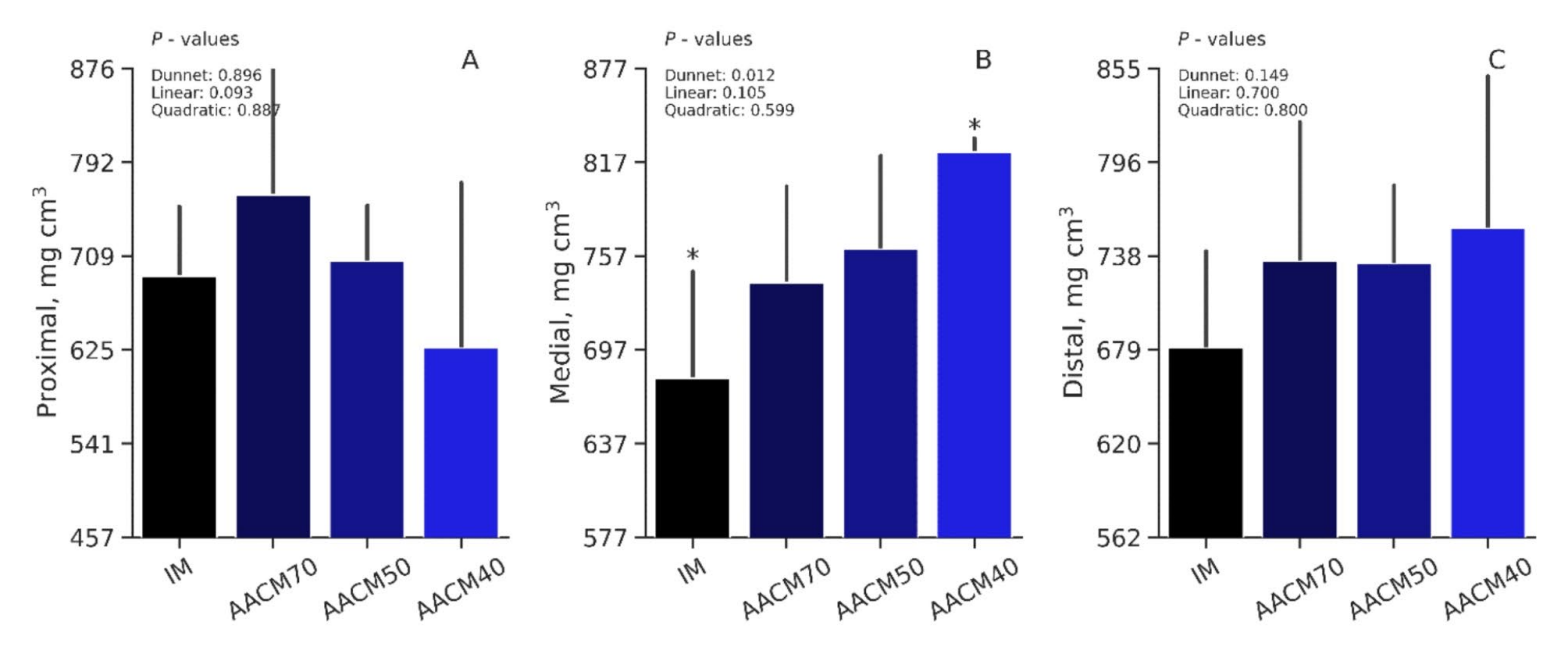
Proximal (**A**), medial (**B**), and distal (**C**) tibial densitometry of eggs from laying hens fed different sources and levels of Zn, Mn, and Cu from 78 to 98 weeks of age.  AACM70 = 42.0, 42.0, 4.9, 28, 0.14, and 1.4 mg/kg of Zn, Mn, Cu, Fe, Se, and I, respectively;  AACM50 = 30, 30, 3.5, 20, 0.10, and 1 mg/kg of Zn, Mn, Cu, Fe, Se, and I, respectively;  AACM40 = 24, 24, 2.8, 16, 0.08, and 0.80 of Zn, Mn, Cu, Fe, Se, and I, respectively. *Differ by Dunnett’s test ( P <  0.05).

No differences between the IM control and AACM treatment groups were detected in the proximal (*P =* 0.90), or distal (*P =* 0.15) tibia bone density (Fig. 2). However, supplementation with AACM was beneficial for enhancing density in the medial segment. Hens receiving diets supplemented with AACM40 demonstrated a higher density, being 21% superior to the IM control (*P* = 0.01). The orthogonal contrast showed no linear or quadratic trends across supplementation levels for the proximal (*P =* 0.88), medial (*P =* 0.60), or distal (*P =* 0.70) segments.

### Intestinal histomorphometry

The intestinal histomorphometry results of the duodenum, jejunum, and ileum are presented in Table 6. In the duodenum, villus height was lower in the AACM70 group compared to the IM control (*P <* 0.01). Similarly, the villus width was narrower in the AACM70 group than in the IM (*P =* 0.02). However, crypt depth (*P =* 0.25), crypt width (*P =* 0.22), villus: crypt ratio (*P =* 0.76), and absorptive area (*P =* 0.44) were not affected by diets. Villus height displayed significant quadratic (*P <* 0.01) trends with supplementation levels, whereas villus width also showed a linear trend (*P =* 0.03).

**Table 6:** Duodenum, Jejunum, and ileum morphometry variables of white laying hens fed different sources and levels of trace minerals from 78 to 98 weeks of age.

Variable	Villus Height (µ)	Villus width (µ)	Crypt depth (µ)	Crypt width (µ)	Villus: Crypt (µ)	Area (µ²)
Duodenum						
IM	1308*	185*	255	68.7	5.12	15.8
AACM70	1131*	161*	236	72.4	5.16	15.3
AACM50	1299	173	252	72.3	5.16	15.5
AACM40	1251	182	237	66.8	5.25	15.0
MEAN	1248	175	245	70.1	5.17	15.4
SEM	14.0	2.9	4.1	1.16	0.086	0.19
*P*-values						
Dunnett	< 0.001	0.015	0.245	0.222	0.757	0.441
Linear^1^	0.002	0.026	0.917	0.084	0.776	0.506
Quadratic^1^	< 0.001	0.226	0.107	0.316	0.539	0.494
Jejunum						
IM	1205*	129*	172*	64.0	7.39	16.7
AACM70	1056*	108*	146*	67.1	7.49	15.4
AACM50	1075	121	139*	62.8	7.52	15.2
AACM40	1015*	127	145*	56.5	7.60	16.0
MEAN	1088	121	150	62.6	7.50	15.8
SEM	20.4	2.4	3.4	1.23	0.178	0.28
*P*-values						
Dunnett	0.006	0.010	0.004	0.060	0.982	0.180
Linear^1^	0.466	0.007	0.884	0.002	0.819	0.396
Quadratic^1^	0.427	0.547	0.435	0.739	0.952	0.439
Ileum						
IM	898	93.0*	164*	58.0*	5.83*	15.3*
AACM70	998	141*	160	73.6*	7.22*	12.7*
AACM50	919	105	138*	63.7	7.41*	15.0
AACM40	887	102	127*	50.7	7.77*	15.9
Average	924	109	148	61.0	7.00	14.7
SEM	17.6	2.6	3.5	1.35	0.23	0.17
*P*-values						
Dunnett	0.112	< 0.001	< 0.001	< 0.001	< 0.001	< 0.001
Linear^1^	0.026	< 0.001	< 0.001	< 0.001	< 0.001	0.242
Quadratic^1^	0.596	0.004	0.526	0.605	0.179	0.841

SEM = standard error of the mean.

IM = 60, 60, 7, 40, 0.2, and 2 mg/kg of Zn, Mn, Cu, Fe, Se, and I, respectively;

AACM70 = 42.0, 42.0, 4.9, 28, 0.14, and 1.4 mg/kg of Zn, Mn, Cu, Fe, Se, and I, respectively;

AACM50 = 30, 30, 3.5, 20, 0.10, and 1 mg/kg of Zn, Mn, Cu, Fe, Se, and I, respectively;

AACM40 = 24, 24, 2.8, 16, 0.08, and 0.80 of Zn, Mn, Cu, Fe, Se, and I, respectively^1^. Orthogonal polynomial contrast; *Within a column differ significantly (*P* < 0.05) according to Dunnett’s test, which compares AACM levels with the IM control group.

In the jejunum, both the AACM70 and AACM40 groups exhibited a smaller villus height compared to the IM control group (*P <* 0.01). However, there was a difference in villus width (*P =* 0.01), with a higher mean in the IM group than in the AACM70 group, whereas AACM40 and AACM50 did not differ from IM. Deeper crypts were observed in all AACM-supplemented groups (*P <* 0.01). However, crypt width (*P =* 0.06), villus: crypt ratio (*P =* 0.98), and area (*P =* 0.18) were not affected by the treatments. Regarding orthogonal contrasts, the histomorphometry of jejunal villus width (*P <* 0.01) and crypt width (*P <* 0.01) was affected by AACM supplementation, with a distinct linear increase in villus width and a decrease in crypt width across different levels of inclusion. Villus height, crypt depth, villus: crypt ratio, and area (*P >* 0.05) did not exhibit any significant linear or quadratic trends.

In the ileum segment, the results indicated effects on all variables except villus height (*P =* 0.11). Villus width in the AACM70 group was greater than that in the IM control group (*P <* 0.01). Crypt depth was significantly lower in the AACM50 and AACM40 groups than in the IM controls (*P <* 0.01). Similarly, crypt width was increased in the AACM70 group (*P <* 0.01) compared to the IM controls. The villus: crypt ratio was also higher in all AACM supplementation groups compared to IM, with the AACM40 group showing the highest ratio (*P <* 0.01). Additionally, there was a decrease in mucosa area, particularly in the AACM70 group, which decreased by 17% compared to the IM group (*P <* 0.01). Ileal villus height showed a linear decreasing trend (*P =* 0.03) with increasing AACM supplementation level. Villus width exhibited a quadratic trend (*P <* 0.01) across the supplementation levels. Both crypt depth and width demonstrated a significant linear decrease with increasing AACM inclusion level (*P <* 0.01). Furthermore, the villus: crypt ratio showed an increasing linear trend (*P <* 0.01). The mucosa area was not affected by the treatments (*P >* 0.05).

## Discussion

The current research supports evidence that supplementation of AACM into laying hen diets leads to improved zootechnical performance, egg quality and bone characteristics when compared to the use of IM. In addition, decreased levels of AACM enhanced egg performance, shell thickness, and tibial density compared with IM treatment. However, excessive inclusion appeared detrimental, with 70% AACM reducing villi width and height. The optimal inclusion of AACM was 40% in relation to IM requirements, which enhanced egg mass, feed conversion, shell quality, and tibial density while maintaining intestinal morphology.

In general, feed intake in monogastric animals is influenced by several factors, including temperature^24^, and the first nutrient limiting in the diet^25–28^. In this study, the average daily feed intake decreased as AACM levels decreased, which implies that the laying hens were regulating their feed intake to meet essential nutrient requirements for optimal zootechnical performance. Nutrient excess or deficiencies regulate the nutritional status via metabolic and physiological processes, as tissues synthesize diverse signaling molecules (neuropeptides, hormones, metabolites) in the peripheral and central nervous systems^29^. These signaling molecules play crucial roles in regulating feed intake, energy balance, and nutrient partitioning throughout the body. For instance, hormones such as leptin and insulin, which are produced in response to nutrient availability, act on the brain to modulate feed intake and energy expenditure. Additionally, neuropeptides, such as ghrelin and neuropeptide Y are involved in stimulating appetite and promoting a positive energy balance when nutrient levels are low^30^. Conversely, other molecules such as cholecystokinin and peptide YY can induce satiety and suppress appetite in situations of nutrient excess^31^. This intricate system of signaling allows the body to maintain homeostasis and adapt to varying nutritional states. Our results conflict with those of Medeiros-Ventura et al.^12^, who found no differences in feed intake among various AACM levels. However, the mechanisms by which these biomolecules precisely regulate the intake of minerals in poultry feed remain unclear, warranting further research.

The AACM supplementation improved egg production, egg weight, egg mass, and feed efficiency in comparison to those laying hens fed IM. The AACM supplementation at a level of 50% showed similar responses to IM, whereas AACM40 resulted in superior improvements in egg mass. The AACM40 hens demonstrated over 6% higher total egg production than IM, indicating optimal mineral intake from AACM. The low bioavailability of IM likely did not promote all the benefits of these trace minerals compared to AACM source. Complexed trace minerals are known to exhibit enhanced absorption and retention in the body, allowing animals to better utilize the minerals to support vital biological processes^6,11^. In the case of egg-laying hens, boosted bioavailability of minerals such as Zn, Mn, Cu, Se, and Fe from AACM supplementation appears to improve performance efficiency^11^. These minerals serve as essential cofactors for enzymes and hormones that regulate oocyte development and ovarian follicle growth. With more robust mineral nutrition, hens can sustain optimal reproductive performance to meet the heavy demands of daily egg production^1,12,32^.

Luteinizing hormones and follicle stimulation are required for the laying process, as they establish and sustain egg production, ovarian follicles, and the ovaries. These gonadotropins attach to receptors in ovarian follicular cells, promoting their development and maturation. Luteinizing hormone stimulates the ovaries, releasing oocytes for fertilization and subsequent egg production^33^. Zinc is required for reproductive hormone production, storage, secretion, and receptor interaction^5^. Additionally, this trace mineral may affect reproductive hormones in mature females^34,35^. Manganese plays a vital role in modulating the hypothalamic–pituitary–gonadal axis, which also regulates these hormones. Adequate Mn levels help optimize gonadotropin production and signaling, follicle recruitment, atresia prevention, and ovulation^36^. Also, Mn is an enzyme cofactor involved in the synthesis of cholesterol, which is the main structural component of ovarian steroids^37^. Manganese deficiency in laying hens results in altered blood levels of ovarian steroids, thereby decreasing reproductive capacity. It has been shown that a diet supplemented with Mn can improve egg production performance in laying hens^38–41^. Further research should investigate the specific mechanisms linking Mn status, hypothalamic-pituitary activity, ovarian steroidogenesis, and follicular dynamics in laying hens. Furthermore, Fe is a crucial element in succinate dehydrogenase activity, thereby enhancing substance metabolism. Because this enzyme plays a pivotal role in energy production through the Krebs cycle^42^, these results indicate that amino acid complexed Fe, as opposed to IM source, supports a higher energy demand for enhanced egg production.

The bioavailability of Zn in laying hen bodies affects egg weight when AACM is supplemented. This essential trace mineral plays a role in the regulation of estrogen and progesterone, both of which are hormones involved in vitellogenesis^34,43,44^. Plasma Mn is an indirect indicator of vitellogenin circulation, a protein precursor that plays a crucial role in egg yolk production and egg quality in laying hens^45^. Similarly, Pereira et al.^11^ reported higher oviduct weights in hens fed AACM. These authors attributed the improvements to a reduction in oxidation and an increase in oviduct cellular integrity.

Albumen height, % albumen, Haugh unit, and yolk color were not influenced by the treatments. In general, an egg consists of three main components: eggshell (9%), albumen (63%), and yolk (27%), and these proportions are determined by the physiology of the hen^46,47^. The lack of differences between treatments for variables related to internal egg quality indicates that both IM and AACM sources provided adequate trace minerals to support albumen development. Nutritional and hormonal signals regulating the complex processes of oocyte growth and nutrient deposition into maturing follicles appear to be unaffected across mineral regimens, even for the highest bioavailability source. These results are in agreement with those of Medeiros-Ventura et al.^12^, who did not find a difference in the albumen of the eggs of laying hens supplemented with different levels of AACM. However, the proportion of egg yolk and shell showed a quadratic trend, with AACM50 having the highest proportion.

Nonetheless, the treatment with 40% trace mineral supplementation resulted in a thicker eggshell. This indicates that an excess of these nutrients may adversely affect the structural formation of the eggshell, with laying hens potentially unable to maintain trace element homeostasis at high dietary levels. Similarly, IM treatment, which is characterized by the low bioavailability of Mn and Zn, was also harmful to eggshell formation. The homeostatic balance observed in the AACM40 treatment indicates a more efficient utilization of Mn and Zn for eggshell synthesis. Indeed, these findings imply that optimal supplemental levels of complexed trace minerals exist that balance availability with production pathways and regulatory mechanisms. Surpassing these finely adjusted requirements disrupts the complex interplay of hormonal, enzymatic, and gene expression, which in turn influences the carbonic anhydrase activity of the shell gland and mineralization dynamics. In fact, Mn is involved in the eggshell structure to regulate the synthesis of glycosaminoglycan and uronic acids in the eggshell membrane, influencing the breaking strength and thickness as well as the modification of the shell ultrastructure^48^. In addition, Zn is an important trace mineral for eggshell production and influences Ca metabolism, shell ultrastructure, and mechanical characteristics^49^. Zinc is a key cofactor of carbonic anhydrase, which converts metabolic CO_2_ to HCO_3_^−^, playing a crucial role in eggshell production. Therefore, Zn availability can directly affect the generation of bicarbonate ions for shell mineralization. The structure and function of carbonic anhydrase can be severely impaired in Zn-deficient hens, which limits HCO_3_^−^ supplies during shell calcification. This phenomenon interrupts crystalline growth and egg development, reducing shell thickness and strength^49^. Beyond carbonic anhydrase effects, Zn also influences receptor-mediated Ca transport from the blood to the eggshell gland. Zinc binds to and activates Ca-ATPase pumps that actively transfer Ca^2+^ across cell membranes for mineral deposition^49^. Zinc deficiency leads to dysfunctional Ca-ATPases, restricting shell mineral matrix production and organization^50^. A Cu deficiency also has a detrimental effect on eggshell quality, as lysyl oxidase enzyme fails to form collagen crosslink proteins, resulting in eggs that are abnormal in size, shape, and shell texture^51^. Studies by Santos et al.^1^ and Medeiros-Ventura et al.^12^ revealed different shell thickness with no differences in the levels of AACM supplementation in laying hens. However, AACM showed thicker shells in comparison to IM and glycinate-mineral supplements, which were considered to have low bioavailability. The physiological state of laying hens and their bone mineral reserves during the study period may explain these differences. Therefore, the ability of hens to improve their shell thickness may depend on their production status and associated metabolic priorities.

Trace mineral deposition in egg yolk for Zn and Fe was not significantly different, implying that variations in mineral supplementation did not lead to differences in the concentrations of these minerals, and AACM50 and AACM40 was equally effective at maintaining Zn and Fe concentrations in the diets, potentially offering cost-effective alternatives to standard IM supplementation. Regarding Mn and Cu deposition, a consistent pattern in the results was observed, with both elements not differing from the IM source, and a linear trend was noted as the levels decreased. The treatment that resulted in lower deposition correlated with higher egg production, indicating that these minerals were in greater demand for forming larger quantities of eggshells with higher thicknesses and maintaining greater medial tibial density. The increased use of Mn and Cu to support higher egg production may have contributed to the observed reduction in egg yolk mineral levels. Overall, our findings show that, even with increased egg production demands on trace mineral supply, the AACM40 diet provided sufficient trace elements to sustain laying rates with improved quality.

In this study, various bone variables were examined across different dietary treatments, and while most of them did not show significant differences, interesting trends in Ca and P content were observed, as well as tibia density. A quadratic trend was observed for bone Ca and P levels, indicating that even at the highest mineral inclusion level (AACM70), these variables did not correlate with the Seedor index, bone strength, and bone density. This divergence from the classical variables used to assess bone quality was particularly evident in bone density, which was higher in the AACM40 treatment. Our findings support the idea that bone density is a more suitable indicator of structural bone quality. This inference is consistent with the results of a previous study by Santos et al.^1^, who found that bone strength, mineral content, and the Seedor index remained relatively consistent across various AACM diets, whereas bone density varied according to supplementation levels. These results highlight the importance of considering not only total mineral content but also bone density when assessing overall bone quality. Furthermore, the lower bone mineral levels observed in the AACM40 group were associated with higher egg production, indicating that minerals stored in laying hen tibias may be used for eggshell formation without negatively impacting bone porosity.

Moreover, our study highlights the role of Zn, Cu, Mn, and Fe in bone quality. Supplementation at 40% of these mineral levels resulted in improved medial density, emphasizing the dependence of bone quality on the ideal and bioavailable amounts of these trace minerals. Research conducted by Richards et al.^3^, Xial et al.^40^, and Zhang et al.^52,53^ demonstrated the involvement of Mn- and Cu-dependent enzymes, such as glucuronyltransferases and lysyl oxidases, in the formation of collagenous bone matrix. Furthermore, Florencio-Silva^54^ noted that Zn-activated enzymes such as phosphatases and carbonic anhydrase play a crucial role in regulating bone deposition and reabsorption. Bones are tissues formed and deformed during the life cycle of a skeleton. Bone homeostasis is a complex process involving osteoblasts that remove and reform bone tissue. Balogh et al.^55^ highlighted the importance of Fe in maintaining the balance between bone formation and deformation. Overloading and a lack of Fe can cause osteoclast activity and bone loss, implying that the optimal level of Fe is crucial for bone homeostasis. Higher Fe concentrations in IM sources may also result in poorer bone quality. This occurs because excess Fe can limit Ca sequestration to mitochondria in the liver and disrupt bone homeostasis. This change is characterized by greater reabsorption induced by osteoclasts and reduced bone production caused by osteoblasts^55^. These findings underscore the vital roles of trace minerals in facilitating essential enzymatic processes critical for bone metabolism and the maintenance of bone structure.

Regarding duodenal morphology, only villus height and width exhibited variations in duodenal morphology among the different treatment groups, indicating alterations in the absorptive surface area of the duodenum. The AACM70 treatment group exhibited lower villus height and width compared to the IM group, potentially denoting a reduced capacity for glucose absorption, as this intestinal segment normally absorbs glucose at a greater rate^56^.

Observations of jejunal morphology across treatment groups revealed variations. Specifically, the villus height in the AACM50 treatment group was consistent with that in the IM group. High dietary AACM levels affected the small intestinal morphology, with the lowest crypt depth observed in the AACM-fed groups. In Dunnett’s test, the IM group exhibited comparatively deeper crypts between treatments, potentially indicating a higher cellular turnover rate in this intestinal segment for maintaining homeostasis. Such increased turnover could impair nutrient digestion and jejunal absorptive capacity. The deeper crypts noted in the IM treatment group imply increased cell proliferation to compensate for cell loss at the villus^57,58^, and this homeostatic process aims to maintain proper digestive and absorptive capacity but requires energy that could divert away from growth and egg production if sustained long-term.

The AACM dietary supplementation induced changes in ileal morphology compared to the IM control. Crypt depth and villus: crypt ratio differed in the AACM groups compared to IM. A higher villus: crypt ratio for AACM40 indicates that these levels of supplementation result in superior ileal morphology, which may have important influences on nutrient absorption. Deeper intestinal crypts contain rapidly proliferating stem cells that differentiate into absorptive enterocytes, which migrate upward along the villus^59^. Therefore, a decreased crypt depth alongside an elevated villus: crypt ratio with AACM inclusion indicates a reduced trace mineral requirement for cellular turnover to maintain the ileal epithelium while still preserving digestive and absorptive capacity^60^. The morphological changes induced by AACM40 supplementation demonstrated that this dosage enhances the structure-function of the ileum.

The improved jejunal and ileal morphology correlates with previously recorded increases in performance and feed efficiency at similar AACM supplementation levels. Consequently, the present morphological findings imply that 40% of AACM supplementation levels induce enterotrophic effects that support intestinal health. Micromineral Zn plays a crucial role in intestinal morphology, influencing both villi growth and the villus-to-crypt ratio, as well as the surface area for absorption^61^. Additionally, Zn directly affects intestinal morphology by increasing cellular proliferation and reducing apoptosis, thereby enhancing absorptive function in the gastrointestinal tract^62^. Trace minerals are key components of antioxidant enzyme complexes, such as glutathione peroxidase, which uses Se as a cofactor, as well as the Cu-Zn superoxide dismutase and Mn superoxide dismutase complexes^63^. Therefore, an adequate supply and appropriate balance of these trace minerals are required to support the activity of the poultry intestinal antioxidant defense system. By maintaining high functionality of endogenous enzymatic antioxidants, the gut barrier can better tolerate inflammatory and oxidative challenges. A study examined the effects of adding Mn to the diet of broiler chickens. The authors found that supplementing feed with Mn appeared to strengthen the intestinal barrier and promote an inflammatory response in the spleen^64^. Given the rapid enterocyte turnover rate in poultry, meeting trace mineral requirements may be pertinent for facilitating the maturation of secreted intraepithelial lymphocytes, which also contribute to intestinal homeostasis^11^. However, excessively high Fe levels can be harmful and provoke an inflammatory response in the gastrointestinal mucosa, as shown by increased lipid peroxidation, cell death, and Fe deposition within intestinal tissues^65^. Consequently, Fe supplementation in poultry diets requires careful regulation to enhance the morphological features of the intestine while preventing adverse effects from Fe overload.

Modulating mineral dynamics in chicken feed formulations is greatly assisted by the addition of phytase enzymes^66^. Furthermore, phytase supplementation may worsen the development of insoluble complexes and increase competition for absorption sites along the intestinal epithelium in diets high in IM. This phenomenon is mainly explained by the hydrolytic action of phytase on phytate, which releases a variety of trace minerals and phytic phosphorus^67^. Also, adsorbents are included in poultry diets for various critical purposes, including mycotoxin binding, ammonia control in poultry houses, improved nutrient utilization, heavy metal sequestration, and moisture management in feed and litter^68^. It has been demonstrated that adsorbents interact with free minerals in the gastrointestinal tract, potentially making these minerals less accessible and insoluble in the intestinal lumen^69^. On the other hand, some of these difficulties might be mitigated if AACM were added to chicken diets. The enhanced stability of AACM is associated with improved mineral bioavailability and reduced interactions with dietary components that could otherwise hinder mineral absorption^5,70^. Notably, the potential binding effects of the adsorbents and the competitive interactions triggered by phytase activity did not significantly affect the efficacy of AACM supplementation in this study. These observations indicate that supplementation of AACM into poultry diets could be an effective strategy for maintaining optimal mineral status, even in environments where mineral bioavailability is typically compromised.

## Conclusion

Replacing IM with lower AACM inclusion levels in laying hen diets improves productive performance, feed efficiency, egg quality, and tibial bone characteristics. The optimal supplementation levels are: 24, 24, 2.8, 16, 0.08, and 0.8 mg/kg of Zn, Mn, Cu, Fe, Se, and I, respectively, corresponding with 40% of IM supplementation requirements, enhancing egg mass, feed conversion, shell thickness, and medial tibia density.

## Data Availability

The data used in this study are available from the corresponding author on reasonable request.
